# Medial Parietal Cortex Activation Related to Attention Control Involving Alcohol Cues

**DOI:** 10.3389/fpsyt.2013.00174

**Published:** 2013-12-20

**Authors:** Thomas E. Gladwin, Mieke H. J. ter Mors-Schulte, K. Richard Ridderinkhof, Reinout W. Wiers

**Affiliations:** ^1^Addiction, Development, and Psychopathology (Adapt) Lab, Department of Psychology, University of Amsterdam, Amsterdam, Netherlands; ^2^EPAN Lab, Behavioural Science Institute (BSI), Radboud University Nijmegen, Nijmegen, Netherlands; ^3^Department of Psychiatry, Academic Medical Center, Amsterdam, Netherlands; ^4^Department of Psychology, Amsterdam Center for the Study of Adaptive Control in Brain and Behavior (Acacia), University of Amsterdam, Amsterdam, Netherlands; ^5^Cognitive Science Center Amsterdam, University of Amsterdam, Amsterdam, Netherlands

**Keywords:** alcohol, attention, medial posterior cortex, fMRI, dual-process, addiction

## Abstract

Automatic attentional engagement toward and disengagement from alcohol cues play a role in alcohol use and dependence. In the current study, social drinkers performed a spatial cueing task designed to evoke conflict between such automatic processes and task instructions, a potentially important task feature from the perspective of recent dual-process models of addiction. Subjects received instructions either to direct their attention toward pictures of alcoholic beverages, and away from non-alcohol beverages; or to direct their attention toward pictures of non-alcoholic beverages, and away from alcohol beverages. Instructions were varied per block. Activation in medial parietal cortex was found during “approach alcohol” versus “avoid-alcohol” blocks. This region is associated with the, possibly automatic, shifting of attention between stimulus features. Subjects thus appeared to shift attention away from certain features of alcoholic cues when attention had to be directed toward their location. Further, activation in voxels located close to this region was negatively correlated with riskier drinking behavior. A tentative interpretation of the results is that risky drinking may be associated with a reduced automatic tendency to shift attention away from potentially distracting task-irrelevant alcohol cues. Future study is needed to test this interpretation, and to further determine the role of medial posterior regions in automatic alcohol-related attentional processes in general.

## Introduction

A complex pattern of attentional biases involving the orienting of attention toward or away from alcohol cues has been found to be related to alcohol use and dependence (1). In a real-life setting, one could imagine an alcoholic walking past a variety of shops and restaurants, but being immediately drawn to a bar, or focusing his or her attention on an advertisement for beer. In laboratory work, the attentional component of such involuntary effects on attention has often been studied using the dot-probe paradigm. In this paradigm, typically a pair of cue stimuli are briefly displayed. After a brief interval a probe stimulus is presented. The probe is presented at the same location as one of the cues. The rationale of the task is that, if attention tends to be drawn to one type of cue, then reaction time and accuracy of responses to the probe will be improved if it appears at such cues’ location. The paradigm has been widely used, for instance to show that anxious individuals have an attentional bias toward angry faces (2). Interestingly, temporal dynamics have been shown to play an important role in such biases: in healthy controls, a fast attentional bias toward threat, found at 100 ms intervals between cues and probe, is followed by a slower attentional disengagement from angry faces at 500 ms (3). Very similar results have been found for faces expressing pain (4). The causal role of attentional biases in problem behavior has been indicated by cognitive bias modification studies, which show that manipulating an attentional bias causes improvements in, e.g., anxiety (5).

Attentional bias studies in alcohol research have shown that, while heavy social drinkers show a bias toward alcohol cues (6, 7), there is evidence for initial orienting toward alcoholic cues but also subsequent attentional disengagement from alcoholic cues has been found in alcohol-dependent subjects (8–10). The initial orienting bias may be related to high incentive salience of drug cues (11–13), which are evidenced by increased neural response to alcohol cues in regions including frontal cortex, the basal ganglia and the limbic system, in individuals at risk of developing dependence and in alcohol-dependent subjects (14–20). The subsequent disengagement may be explained by slower, reflective processes that result in an attentional shift away from temptation.

In typical cue reactivity or dot-probe tasks, a conspicuously lacking task feature is that of conflict. The probability of a probe appearing at the biased location is as good as it appearing at the neutral control location. Subjects may therefore have little reason not to allow their attention to be modulated: it will help as often as it hinders. This may be important in interpreting results of the studies using the task, as from the perspective of dual-process models the essential feature of addiction is an imbalance between reflective and impulsive processes (21–24), rather than the existence of strong automatic appetitive reactions alone. A task that is designed to evoke a conflict between impulsive and reflective processes related to attentional shifting may therefore provide complementary information on the neural mechanisms underlying attentional biases related to alcohol.

In the current study, we developed such a task in order to study neutral activation underlying attentional biases in this reflective-versus-automatic context. As yet, relatively little is known about this. However, studies on voluntary and stimulus-driven attentional mechanisms (25–27) suggest two possibilities. If subjects have an engagement bias toward alcohol stimuli, activation in frontoparietal regions related to the controlled shifting of attention would be expected when subjects need to direct their attention away from alcohol cues. In contrast, a disengagement bias would be expected to lead to increased activation in regions related to spatial or non-spatial attentional shifting, e.g., the precuneus (28, 29), when directing attention toward alcohol cues. Note that although such automatic disengagement might not appear to be in line with task instructions, shifting attention away from alcohol-related stimulus features could work to avoid distraction from the task by the alcohol-related content of cue. Supporting this possibility, in the context of smoking dependence the presence of smoking cues has been found to evoke brain activation when such cues could potentially distract from a task being performed (30). To the aim of exploring these hypotheses, subjects performed a cueing task during functional magnetic resonance imaging (fMRI). Further, activation associated with attentional approach-avoid conflicts was correlated with individual differences in drinking behavior, which may provide suggestions about which alcohol-related cognitive processes underlie risky drinking.

## Materials and Methods

### Participants

Thirty-five subjects participants were recruited from a student population (mean age 21, 28 female). Inclusion criteria were an AUDIT-score above zero (range: 1–21; *M*: 7.97; SD: 4.66), comprehension of the Dutch language, and right-handedness based on the Edinburgh Handedness Inventory (31). An AUDIT-score above zero was required in order to exclude subjects who never drink alcohol. All participants signed an informed consent form prior to the experiment, and were compensated for their participation by course credits or monetary rewards.

### Procedure

Before scanning, subjects performed a computerized preference test in order to select their preferred alcohol beverages and soft drinks as relevant stimuli. Subjects were presented with 22 pictures of beverages (11 alcoholic beverages and 11 soft drinks), which were presented in pairs of two presented next to each other. They had to choose the beverage they drank most often, by pressing the left or right response key corresponding to their selection. The four alcoholic beverages and the four soft drinks with the highest preferences were used during the cueing paradigm. Participants then briefly practiced the cueing task (see below) to make sure they understood the task instructions. Subjects subsequently performed the task in the scanner for 20 min. Following the task, subjects filled out the alcohol use disorder identification test [AUDIT (32)], an internationally validated screening instrument for alcohol use and problems. The test is routinely used to screen individuals with likely alcohol-problems in diverse, clinical and non-clinical, contexts. The test identifies patterns of “excessive” or hazardous drinking, either in clinical or non-clinical samples (32). Across several studies, alpha coefficients were approximately 0.80 (33), indicating good internal consistency. Furthermore, the AUDIT was able to identify persons with harmful/hazardous alcohol consumption in 92% of the cases (32).

### Task

**Figure 1:**
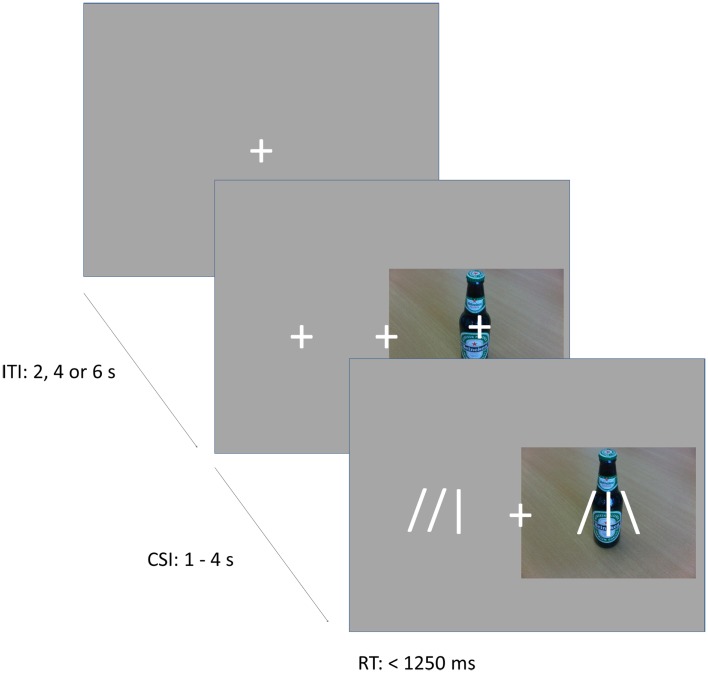
**Illustration of the task**. The figure shows an example of valid probe trial in an approach alcohol block, or an invalid probe trial in an avoid-alcohol block. The probe appears at the location of the cue, requiring an “up” response.

In the cueing task (Figure 1), subjects were instructed to direct their attention either toward or away from the locations of pictures of alcoholic and soft drinks, which were randomly presented to the left or right of a fixation cross. Instructions were varied over blocks in pseudo-random order, and were provided by an 8 s instruction period before each block. In alcohol-approach blocks, the instructions were to direct attention toward alcoholic drinks and away from soft drinks. In alcohol-avoid blocks, the instructions were to direct attention toward soft drinks and away from alcoholic drinks. The task existed of 10 blocks, each consisting of 20 trials. Trials started with a fixation cross in the center of the screen. The fixation cross was presented for 2 s (on 1/2 of the trials), 4 s (on 1/3 of the trials), or 6 s (on 1/6 of the trials), to provide jitter for event-related fMRI analyses. Following the fixation period, two additional crosses appeared to the left and right of the central fixation cross and a picture of an alcoholic beverage (Alcohol trials) or soft drink (Soft drink trials) appeared centered behind either the left or right cross. These stimuli remained onscreen for 1–4 s. Depending on block type and picture type, subjects directed their attention either to the position of the picture (Approach trials), or to the opposite position (Avoid trials). On 25% of all trials, a probe appeared after the stimulus period to which subjects had to respond. The probe consisted of an abstract arrow pointing up or down (the symbols 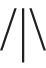 or 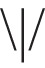), and was located at the attended position on 80% of the trials (Valid Cue trials), and on the opposite side in the remaining 20% of trials (Invalid Cue trials). The 80% probability of valid cueing was used to reinforce shifting attention as required by the task. Participants had to indicate the orientation of the arrow by pressing one of two, “up” and “down,” response buttons. Opposite to the probe a distractor stimulus was presented consisting of similar visual features (e.g., 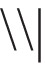). This was done to reduce the pop-out effect of a single probe and hence increase the importance of correctly shifting attention.

### fMRI data acquisition

Functional magnetic resonance imaging data were collected using a Philips Achieva XT 3T head-only MRI scanner at the University of Amsterdam, Spinoza Center. Participants were scanned in a supine position, using a 32 channel SENSE head coil. Ear plugs and head phones were used to reduce scanner noise and to communicate with the subjects. Foam padding was used to stabilize head position and minimize head movement. Stimuli were presented using Presentation software running under Windows XP operating system projected onto a back-projection screen, which was placed at the head of the scanner. This could be viewed by looking into a mirror attached to the head coil, and could be adjusted individually to maximize viewing comfort. A MRI compatible fiber optic response device (fORP) with a five-button paddle was used for responses, of which subjects only had to use the buttons under their index and middle finger. Functional scanning was synchronized with the beginning of the experiment through a trigger pulse sent by scanner to the presentation software.

For each subject, a structural T1-weighted echo planar imaging volume (TR = 8.1 ms; TE = 3.7 ms; matrix size = 256; 160 sagittal slices 1 mm thick; 0 mm gap; field of view = 256, 256, 160; SENSE factor = 1) was obtained prior to the paradigm. Functional imaging was performed using an echo planar gradient imaging sequence and transversal orientation. The following parameters were used in order to obtain these scans: 605 scans, TR = 2000 ms; TE = 27.63 ms; matrix size = 64; 37 transversal slices 1 mm thick; 0.3 gap; field of view = 192, 192, 122; SENSE factor = 2.5.

### Behavioral data analysis

Reaction time and accuracy were analyzed of those trials on which a probe appeared to which a subject could respond. Note that behavioral analyses are limited due to the relatively low number of response trials in the current fMRI paradigm; the analyses are primarily intended to check whether subjects’ behavior was as expected. Trials with reaction times outside the range of 150–3500 ms were rejected, as were trials on which the incorrect response was given. The following task conditions were used a factors. First, Invalid versus Valid cueing trials were distinguished: in valid trials, the subject had been instructed to direct their attention to the location of the cue. In invalid trials, the instructed shift in attention was to the incorrect location, and therefore would have to shift their attention of probe presentation. Second, the stimulus type of the picture used as a cue was a factor: was the picture of an alcoholic drink or a soft drink. Finally, the instructed attentional shift was a factor: did subjects have to shift their attention toward or away from the cue. Reaction times and accuracy were analyzed using a repeated measures ANOVA with Validity (invalid or valid cueing), alcohol (soft drink picture or alcohol picture), and approach versus avoid as within-subjects factors, and *AUDIT*-score as between-subjects factor.

### fMRI data analysis

The data were analyzed using statistical parametric mapping (SPM8; Wellcome Department of Cognitive Neurology, London, UK). Images were corrected for differences in slice time acquisition and motor corrected using rigid body transformation parameters. Functional images were coregistered to the anatomical scan, and spatially normalized to a T1 template based on the Montreal Neurological Institute (MNI) stereotaxic space. Normalized functional images were interpolated to 3 mm cubic voxels. Functional images were spatially smoothed with a Gaussian filter (8 mm full width – half maximum). A high-pass filter with a cutoff period of 128 s was applied to functional images to remove slow signal drifts.

Event-related responses were used as predictors for the trials of the two block types. These responses were modeled as a hemodynamic response function convolved with stick functions, placed at the time points of each trial’s cue presentation: that is, the appearance of the picture that, together with the block instructions, instructed subjects to shift their attention toward or away from the picture location. Valid and invalid probes and instruction periods were modeled as nuisance effects, based on stick functions and boxcar functions, respectively.

A contrast was calculated as the difference between regression weights for the two block types: approach-alcohol (and avoid-soft drink) minus avoid-alcohol (and approach-soft drink). Further, correlations between individual contrast scores and AUDIT-scores were tested. Statistical maps were thresholded at *p* = 0.005, 20 voxel cluster size minimum; we acknowledge that this may be considered liberal, but consider this to be a reasonable compromise between power and false positive in fMRI data (34), especially for exploratory studies. Activation outside gray matter (e.g., in ventricles, possibly reflecting respiratory artifacts) was excluded from further analysis.

## Results

### Behavioral results

Subjects shifted their attention as instructed, as shown by significant effects of valid versus invalid cueing on reaction time [*F*(1, 34) = 61.7, *p* < 0.0001, $\eta_{p}^{2}$ = 0.65; participants were faster when the cue was valid, 931.23 ms, compared to when the cue was invalid, 1314.02 ms] and on accuracy [*F*(1, 34) = 5.43, *p* < 0.03, $\eta_{p}^{2}$ = 0.14; participants responded more accurately when the cue was valid, 97%, compared to when the cue was invalid, 92%]. Further, an interaction between validity and alcohol cue was found [*F*(1, 34) = 9.51, *p* < 0.004, $\eta_{p}^{2}$ = 0.210]: for invalid trials only, responses were significantly faster following alcohol than non-alcohol cues. That is, subjects were faster to shift their attention to an invalidly cued location following an alcoholic versus a non-alcoholic cue. No other main effects or interactions were significant, which could have been due to the infrequent occurrence of probes.

### fMRI results

**Figure 2:**
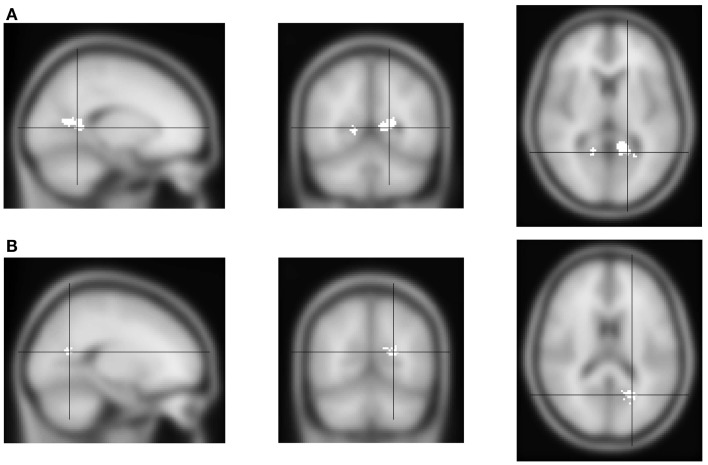
**FMRI activation for the “toward alcohol” – “away from alcohol” contrast**. From left to right, sagittal, coronal and transversal views, left and right reversed. **(A)** Activation of the contrast “toward alcohol” versus “away from alcohol” blocks. Significant voxels are shown in white. Activation was found in medial parietal cortex, near the precuneus and posterior cingulate. **(B)** Interaction of the block contrast with AUDIT-scores: white voxels show the region where contrast values were significantly lower for individuals with higher AUDIT-scores. Activation was again found in medial parietal cortex, but somewhat more posterior.

First, we analyzed the fMRI contrast between block types: attend alcohol minus avoid-alcohol. Positive effects would therefore indicate increased activation when subjects are instructed to direct their attention toward alcohol pictures, and therefore are focusing on a potential distractor. Negative effects would indicate increased activation when subjects have to disengage from a theoretically attractive cue, which might require the involvement of additional control networks. The results showed only bilateral activation in a medial posterior region, which could be described as the junction of the calcarine and parieto-occipital sulcus; or of the precuneus and the posterior cingulate (Figure 2A, Table 1).

**Table 1:** **FMRI results**.

	No. of voxels	Extreme *t*	*X* (mm)	*Y* (mm)	*Z* (mm)
Block type	25	3.5	16	−54	6
	283	4.2	−18	−52	10
Block type × AUDIT	80	3.7	−20	−62	18

*Regions of activation. Block type: the contrast between attend alcohol and avoid-alcohol blocks. The interaction with AUDIT-scores is represented by “Block type × AUDIT.” No. of voxels: number of adjacent voxels above threshold (*p* < 0.005). Extreme *t*: highest *t-*value in the region of activation. *X, Y*, and *Z* (mm) are the coordinates of the center-of-mass in MNI space*.

Second, we analyzed the correlation between individuals’ activation for the above block-related contrast, and their AUDIT-scores. This would indicate the extent to which more versus less hazardous drinkers show activation related to the Approach- versus Avoid-Alcohol block types. An interaction between the contrast and AUDIT-scores was found in only one region, close to the location of activation found for the independent within-subject analysis: AUDIT-scores were negatively correlated with this activation (Figure 2B, Table 1). This indicates that more hazardous drinkers showed a weaker Approach-Alcohol-related activation of the medial parietal region.

## Discussion

Subjects performed a task in which, on every trial, they were instructed to direct their attention toward or away from the location of an alcoholic or non-alcoholic cue; a task-relevant stimuli would usually appear at the attended location. Depending on the involvement of automatic engagement or disengagement processes, the instructed (i.e., controlled) behavior could lead to alcohol-related conflict or distraction. The results suggest that medial parietal cortex, or precuneus, may play a role in such situations. This region has been associated with self-referential processing, encoding spatial relations for body movement control, and the shifting of attention (28), in particular shifts of attention between stimulus features (29). In the current study, this region was activated when attention had to be directed toward rather than away from alcoholic cues, and riskier drinkers showed a reduction in this activation. Subjects therefore appeared to shift attention away from alcohol-related stimuli dimensions when preparing to respond to a probe at the same location. This would be in line with a role of disengagement: subjects may shift attention from certain stimulus features, to avoid distraction due to instructions demanding their spatial attention be directed toward the alcohol cue. These neural processes may have been automatically activated: despite the association of the medial parietal cortex (precuneus) with conscious attentional shifting in much of the literature, it may also be involved in implicitly cued shifts of attention (35).

Thus, a tentative interpretation of the current results is that the activation of attentional shifting mechanisms when confronted with an alcohol cue reflects a tendency to inhibit the representation of stimulus features with high incentive salience (12), when they could interfere with task performance. In that case, the decrease of activation as drinking becomes more risky may be a factor in the loss of control over drinking. If some individuals indeed have a weaker “protective” tendency to shift attention away from potentially distracting alcohol cues they may be more vulnerable to stimuli that increase the likelihood of drinking, or decrease the likelihood of applying control over the impulse to drink.

We note a number of limitations of the current study. Subjects were not recruited from a clinical population, which could limit the generalization of the results of this study due to differences from patients in terms of the cognitive processes underlying addictive behavior as well as other variables such as age, living condition, and cognitive impairments. Second, the proportion of males and females differed for more and less hazardous drinkers: there were few male non-hazardous drinkers in the current sample [a general feature of the Dutch student population (36)]. This could have resulted in a differential contribution of gender within the two subject groups. In addition, a behavioral task optimized for fMRI was used, leading to a limitation of behavioral data. In future research a behavioral task could be performed outside the scanner to obtain more detailed behavioral correlates of attentional bias. Furthermore, this study only assessed hazardous drinking by use of the *AUDIT* questionnaire. While we note that this questionnaire is commonly used to characterize excessive or hazardous drinking in non-clinical populations, more measures should be used in future research. Of particular interest would be assessments of craving, such as the craving typology questionnaire [CTQ (37)]. Processes related to attention and attentional shifting may well be related to craving or specific components of craving, e.g., reward, relief and obsession (37). Inclusion of these measures will provide a better characterization of the sample, and a more comprehensive view of contributing factors in the development and/or the maintenance of addiction. Finally, more research is needed to establish the precise cognitive role of the precuneus in attentional biases involving alcohol stimuli. Our current interpretation of the data rests on reverse inference (38): we attempt to use effects of manipulations of cognition on brain activation to infer cognitive effects from brain activation. We agree that such inferences should always be considered uncertain, playing a role as a step in a process of convergent understanding of brain and cognition. The validity of our interpretation of the current results is clearly dependent on, first, the replication of the results themselves, as well as the degree to which alternative explanations could play a role. For instance, perhaps information processing of peripherally located stimuli differs dependent on task load. Further research is needed to explore such possibilities.

Based on the current findings, medial parietal cortex should be considered as an *a priori* region of interest in future studies. Further, if it turns out that attentional shifting indeed plays a protective role in drinking behavior, this may generate hypotheses for mediating factors for interventions (39, 40) and suggest methods to screen for risk of addiction. In particular, the results suggest a more specific target for attentional retraining than attentional control in general; e.g., perhaps subjects could be effectively trained to automatically shift attention away from alcohol-related stimulus features, and thereby be less vulnerable to cue-evoked behaviors. Further study of brain activity in the context of conflict between explicit task demands and alcohol-related biases would appear to be of interest, in particular in clinical groups.

## Conflict of Interest Statement

The authors declare that the research was conducted in the absence of any commercial or financial relationships that could be construed as a potential conflict of interest.
